# Tribocorrosion Behavior of NiCoCrMoCu Alloys Containing Different Carbides in Acidic Media at Various Applied Loads and Sliding Speeds

**DOI:** 10.3390/ma17122971

**Published:** 2024-06-17

**Authors:** Chao Li, Ziming Zeng, Jianwei Teng, Biaobiao Yang, Yunping Li

**Affiliations:** 1 State Key Laboratory for Powder Metallurgy, Central South University, Changsha 410083, China; lichao97@csu.edu.cn (C.L.); zndxzzm@csu.edu.cn (Z.Z.); biaobiao.yang@imdea.org (B.Y.); 2 Institute for Advanced Studies in Precision Materials, Yantai University, Yantai 264005, China; 3 IMDEA Materials Institute, C/Eric Kandel 2, Getafe, 28906 Madrid, Spain; 4 Department of Materials Science, Polytechnic University of Madrid/Universidad Politécnica de Madrid, E.T.S. de Ingenieros de Caminos, 28040 Madrid, Spain

**Keywords:** NiCoCrMoCu alloy, dry sliding wear, tribocorrosion, carbide reinforcement, metal matrix composites

## Abstract

In this study, the ball-on-disk sliding wear and tribocorrosion behavior in the H_2_SO_4_ and HCl solution of NiCoCrMoCu alloys with carbon additions of 0.2, 1, 1.5, and 2 wt.% with the Al_2_O_3_ ball as a counterpart was investigated systematically. Obvious tribocorrosion antagonistic effects were found after wear in both aqueous solutions. Compared with dry sliding wear conditions, the lubrication effect of the aqueous solution significantly reduces the wear rate of the alloy, and the reduction effect in the H_2_SO_4_ aqueous solution was more obvious than that in HCl. The antagonistic effects of the 0.2C and 1C alloys decrease with the load and sliding rate, while those of the 1.5C and 2C alloys increase. The (coefficient of friction) COF and wear rate under different loads and sliding rates were analyzed using the response surface analysis (RSM) method. It was found that the COF mainly showed dependence on the sliding rate, while the wear rate showed dependence on load and sliding speed.

## 1. Introduction

Ni-based alloys are used as engineering materials to withstand stress corrosion [1,2,3] due to their excellent mechanical properties [4,5] and harsh environment resistance [6,7,8]. The γ′ precipitated phase in the Ni-based alloy is embedded in the γ matrix in a coherent or semi-coherent structure [9]. Due to the limited lattice mismatch [10], the coherent distortion between γ′ and γ has a limited hindering effect on dislocation movement [11,12]. The current shortcoming of the Ni-based alloy is its low wear resistance caused by insufficient hardness [13]. Considering the high cost of Ni alloys, it is very economically feasible to improve the wear resistance of Ni-based alloys.

Introducing high-density and non-deformable particle phases into the matrix is an effective method to improve wear resistance [14,15], which can reduce material removal by reducing the contact area between the matrix and the counterpart and inhibiting the plastic deformation of the matrix [16,17]. The particle phase in the composite can be formed by an in situ reaction of element doping or added by external addition [18], among which the in situ precipitation particle phase [19] is small in size, evenly distributed, has good metallurgical bonding with the matrix, and is more beneficial to wear resistance. Carbide is a common particle phase in Ni-based alloys and is formed in situ by adding a small amount of C [4,20]. Ye et al. [21] reduced the wear rate of the CoCrFeNiMn alloy by 78% by precipitating Cr_7_C_3_ in situ through the powder metallurgy method, in which the C element was added in the form of graphene. Gao et al. [22] fabricated in situ carbide-reinforced Ni-based composites by hot-pressing sintering Ni and Ti_2_AlC powders; the friction coefficient and wear rates of composites reduce with increasing Ti_2_AlC content, especially for the wear rate.

However, the introduction of carbides is generally detrimental to the corrosion resistance and tribocorrosion performance of the alloy. The in situ precipitation of carbides will consume the key corrosion-resistant elements Cr and Mo in the matrix [23], and the potential difference between the carbide and the matrix will increase the local corrosion tendency of the alloy [24]. The tribocorrosion process [25] is more complex than the mechanical wear process because this process includes the mechanical wear, corrosion, and interaction of corrosion and wear on the material, which has been summarized in the Mischler [26] by the wear accelerated corrosion model and Archard [27] by the mechanical wear model. In the above models, tribocorrosion modeling focused on the chemical and mechanical factors in the two-body contact system without considering the lubrication effect. Cao et al. [28] proposed the tribocorrosion of passivated metal under a mixed lubrication state and used this theoretical model successfully in metal-on-metal artificial hip joints. Zhu et al. [29] found that the material loss induced by tribocorrosion was much less than that by mechanical wear in the tribocorrosion process of a Monel 400 alloy in seawater, nano-Cu particles were formed in situ at the surface under tribocorrosion due to the preferential corrosion of Ni, and Cu particles enhanced the lubrication effect of the tribolayer and reduced the wear rate.

External conditions, such as load and the sliding rate, have a great influence on the tribocorrosion properties of alloys. Due to the lubrication effect of aqueous solutions, Gao et al. [30] found that the wear rate first increased and then decreased with load during the tribocorrosion process of SAF 2205 Duplex Stainless Steel in Artificial Seawater, which is different from the monotonous increase in the wear rate with load in dry sliding wear. Sun et al. [31] and Namus et al. [32] observed the phenomenon that the wear rate increases with the sliding rate in the tribocorrosion of stainless steel and Ti6Al4V, respectively, and the increase in sliding rate will intensify the pitting corrosion behavior of the alloy and the passive film removal rate. NiCoCrMoCu has been proven to have excellent corrosion resistance in acidic [33,34] and neutral [35] solutions, even after severe deformation. However, there is currently a lack of detailed research on the tribocorrosion performance and mechanism of the NiCoCrMoCu alloy under different media and external parameter conditions.

In this study, we conducted tribocorrosion experiments under different loads and sliding rates on the carbide-strengthened NiCoCrMoCu alloy in oxidizing and reducing media, and dry sliding wear experiments were conducted under the same parameters for comparison. The effect of the addition of C content on the wear behavior and wear mechanism of the NiCoCrMoCu alloy under different conditions and different parameters was analyzed. We used the response surface analysis (RSM) [36,37] method to analyze the experimental results, which can enumerate the correlation between input parameters and output variables with the minimum number of experiments and is very suitable for establishing the relationship between wear behavior and parameter variables in wear experiments.

## 2. Experiment

### 2.1. Materials

The nominal compositions of the four NiCoCrMoCu alloys prepared by powder metallurgy are shown in Table 1. According to the addition of C content, they are named the 0.2C, 1C, 1.5C, and 2C alloys. Alloy powder with a particle size of less than 150 μm is collected and formed by hot isostatic pressing (HIP). The parameters are insulation and pressure holding at 1200 °C and 155 MPa for 4 h.

### 2.2. Phase Identification

To accurately analyze the types of carbides in different alloys, we extracted the carbides in the 0.2C, 1C, 1.5C, and 2C alloys through the standard extraction method of ASTM-E963-95 [38]. The extraction solution is hydrochloric acid and methanol with a volume ratio of 10:1. The extracted carbides were dried and then subjected to X-ray diffraction (XRD) phase identification, and the equipment and experimental parameters used in XRD experiments are summarized in Section 2.5.

### 2.3. Mechanical Behavior

The hardness of the 0.2C, 1C, 1.5C, and 2C alloys was measured by a hardness tester (200HV-5, Laizhou, China) with a loading force of 2 kg and a holding time of 15 s. Each alloy was tested ten times, and the hardness result was the average of the data after removing the maximum and minimum values. The compression property of the HIP and HEX alloy was performed by a universal material testing machine (INSTRON 5982, Norwood, MA, USA). The compression rate was 10^−3^ m/s, and each test was repeated three times.

### 2.4. Dry Sliding Wear and Tribocorrosion Tests

A ball-on-flat wear testing machine (MWF-002, Jinan, China) was used to conduct dry sliding wear and tribocorrosion experiments on the 0.2C, 1C, 1.5C, and 2C alloys. The sliding time, load, and sliding rate were consistent, and the corrosion medium was 0.5 mol/L H_2_SO_4_ and 1.2 mol/L HCl aqueous solutions. The sliding time is 3600 s, the load is 30, 50, and 70 N, and the sliding speed is 75, 150, and 225 mm/s. The counterpart is an alumina ball with a diameter of 6 mm, and the Vickers hardness is 1600 HV. To ensure the accuracy of the experiment, three experiments were conducted under each set of parameters. The wear rate of the alloy can be calculated according to the following formula:

(1) $$\omega =\frac{V}{L\times P}$$

where V is the wear volume loss (mm^3^), L is the sliding distance (m), and P is the applied load (N). V is obtained by dividing the wear mass loss by the alloy density.

As shown in Formula (2) [39,40], the wear rate T in tribocorrosion can be divided into three parts as follows:

(2) $$T=W_{0}+C_{0}+S$$

where *W*_0_ is the mechanical wear rate, *C*_0_ is the corrosion rate_,_ and *S* is the synergistic effect of corrosion and wear on the wear rate.

### 2.5. Characterization of the Microstructure

X-ray diffraction (XRD, Smart lab 3 kW, Tokyo, Japan) was used to identify the phases in the alloy and the extracted carbides. XRD measurements were conducted from 30 to 100° with a scanning rate of 8°/min. Initial microstructures, wear surface morphology, and element distribution of the 0.2C, 1C, 1.5C, and 2C alloys were characterized by a scanning electron microscope (SEM) and an energy-dispersive spectrometer (EDS) using a field emission scanning electron microscope (SEM; FEI Quanta 650; Waltham, USA). The effect of carbon addition on grain size was analyzed using electron backscattered diffraction (EBSD) technology with a voltage of 30 kV and a step size of 0.1 μm. Further EBSD analysis was performed with HKL Channel 5 software (https://nano.oxinst.cn/products/ebsd/post-processing-software, accessed on 13 June 2024) (HKL Technology Ltd., Hobro, Denmark). A laser scanning microscope (LSM, S neox 090, Barcelona, Spanish) was used to characterize the width and depth of the wear surface.

## 3. Results and Discussion

### 3.1. Microstructure of the NiCoCrMoCu-xC Alloy

**Figure 1:**
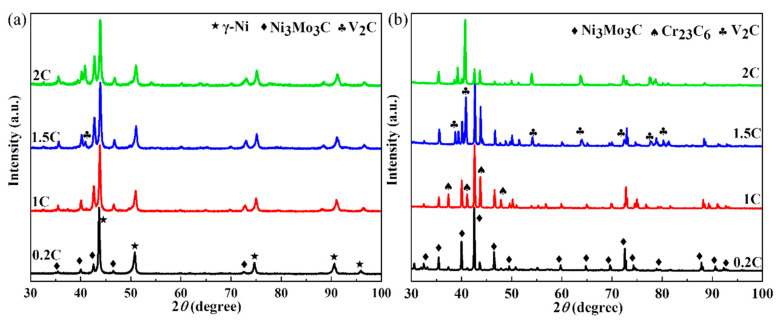
The XRD results of (**a**) the HIPed alloy and (**b**) extracted carbide.

Figure 1 shows the XRD results of the hot isostatically pressed alloy and the carbides in the alloy after extraction. As can be seen in Figure 1a, there are Ni matrix peaks and Ni_3_Mo_3_C peaks in the 0.2C and 1C alloys. As the C content increases, V_2_C peaks are detected in the 1.5C and 2C alloys. Figure 1b shows that the Ni_3_Mo_3_C peak intensity is the highest among the 0.2C, 1C, and 1.5C alloys, while the V_2_C peak intensity is the highest in the 2C alloy. The changes in the corresponding carbide types of the highest peak indicate the existence of transitions between carbides. It is worth noting that the peak of Cr_23_C_6_ was detected in the carbide after extraction for the 1C, 1.5C, and 2C alloys.

**Figure 2:**
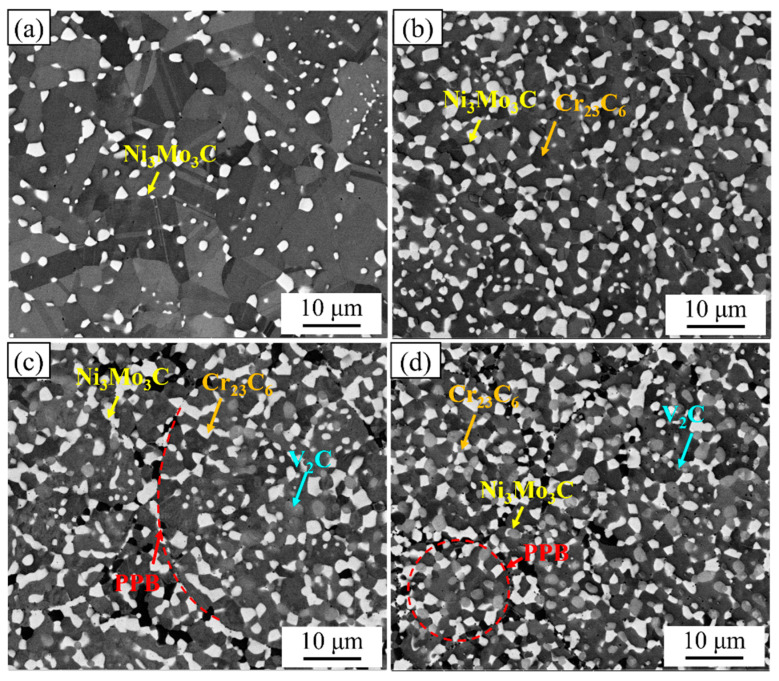
The initial microstructures in backscatter diffraction mode: (**a**) 0.2C, (**b**) 1C, (**c**) 1.5C, and (**d**) 2C.

Figure 2 shows the initial microstructure of the 0.2C, 1C, 1.5C, and 2C alloys in BSE mode. It can be seen that there are white, black, and gray carbide phases, which we have identified in previous studies as Ni_3_Mo_3_C, Cr_23_C_6_, and V_2_C, respectively [41]. Image-J (https://imagej.nih.govij/ (accessed on 13 June 2024)) software was used to count the size and phase fraction of carbides in the alloy, and the results are summarized in Table 2. The sizes of the Ni_3_Mo_3_C phases in the 0.2C, 1C, 1.5C, and 2C alloys are 1.03, 1.39, 1.42, and 1.08 μm, respectively, and their phase fractions are 9.5, 25, 23.5, and 17.3%, respectively. The size and phase fraction of the Ni_3_Mo_3_C ffswfswephase showed a trend of first increasing and then decreasing, and the changing trends were not completely synchronous. The peaks of the size and phase fraction appeared in the 1.5C and 1C alloys, respectively. The sizes of the Cr_23_C_6_ phases in the 1C, 1.5C, and 2C alloys are 1.77, 0.67, and 0.63 μm, respectively, and their phase fractions are 10, 5.8, and 6.1%, respectively. The size and phase fraction of the Cr_23_C_6_ phase both show a monotonically decreasing trend. The sizes of the V_2_C phases in the 1.5C and 2C alloys are 0.7 and 0.73 μm, respectively, and their phase fractions are 11.8 and 17.8%, respectively. Both the size and phase fraction of the V_2_C phase show a monotonically increasing trend. Overall, as the C content increases, the Ni_3_Mo_3_C phase and the Cr_23_C_6_ phase tend to transform into the V_2_C phase. It is worth noting that the presence of prior particle boundaries (PPBs) was observed in the 1.5C and 2C alloys, which is a common defect in powder metallurgy and is detrimental to mechanical properties [42].

**Figure 3:**
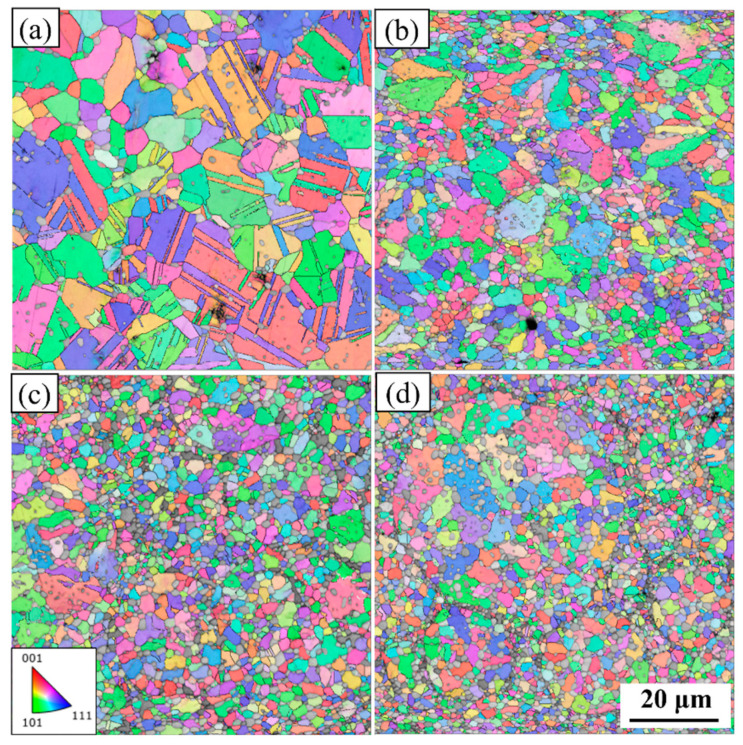
Inverse pole figure superimposed with a band contrast of (**a**) 0.2C, (**b**) 1C, (**c**) 1.5C, and (**d**) 2C.

Figure 3 shows the inverse pole figures (IPFs) superimposed with the band contrast of the 0.2C, 1C, 1.5C, and 2C alloys. The colored parts in the picture are grains and the gray parts are unresolved carbides. The average grain sizes of the 0.2C, 1C, 1.5C, and 2C alloys are 2.6, 1.8, 1.6 and 1.4 μm. The addition of C content can effectively refine the grains by precipitating carbides in situ [43]. A large number of twins can be observed in the 0.2C alloy, which is due to the reduction in stacking fault energy caused by the higher Co content [44]. Carbides in the 0.2C and 1C alloys are distributed at grain boundaries and grain boundary intersections, which can effectively inhibit grain boundary sliding and grain growth and improve the strength of the alloy. Carbides in the 1.5C and 2C alloys exist in large quantities at grain boundaries and PPBs.

### 3.2. Mechanical Behavior of the NiCoCrMoCu-xC Alloys

**Figure 4:**
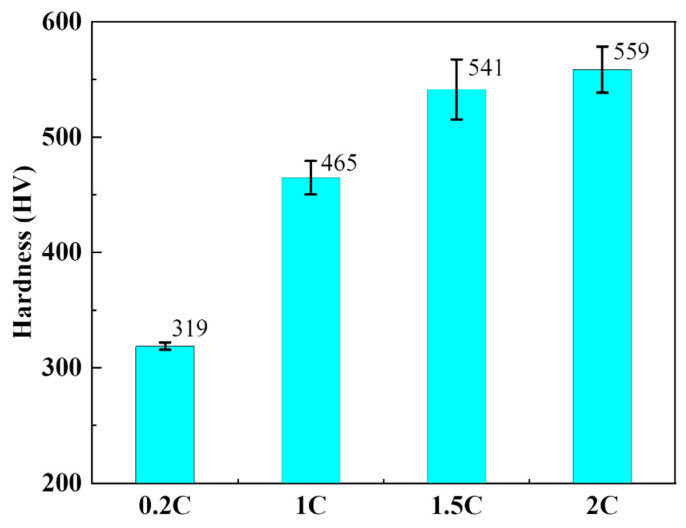
Vickers hardness of the 0.2C, 1C, 1.5C, and 2C alloys.

As shown in Figure 4, the Vickers hardness values of the 0.2C, 1C, 1.5C, and 2C alloys are 319, 465, 541, and 559 HV, respectively. The addition of C content can significantly increase the hardness of the alloy. Table 3 summarizes the yield strength and ultimate compressive strength of the 0.2C, 1C, 1.5C, and 2C alloys. For convenience of comparison, the maximum contact stress during the wear process is also placed in the table, and the calculation results are based on the Hertzian contact stress method [45]. The yield strength of the alloy increases significantly with the C content, which is mainly due to the precipitation of carbides that increases the plastic deformation resistance in the alloy and the refinement of the grains [46]. The elongation drops from 45% in the 0.2C alloy to 20% in the 2C alloy. The decrease in elongation is due to the existence of carbides and original particles. Dislocation accumulation is prone to occur at the interface between carbides and the matrix, leading to stress concentration cracking. The original particle boundaries composed of carbides are often the source of crack initiation. These two reasons lead to a significant decrease in elongation. It is worth noting that the 0.2C alloy has the highest compressive strength of 2287 MPa among all alloys, which comes from its excellent work-hardening ability. We can also see in Table 3 that, except for the 0.2C alloy under a 30 N load, the maximum contact stress of the alloy under all loads exceeds the ultimate compressive strength of the alloy. It can be expected that cracks will appear in the alloy during wear.

### 3.3. Friction Coefficient and Wear Rate of the NiCoCrMoCu-xC Alloys in Various Conditions

**Figure 5:**
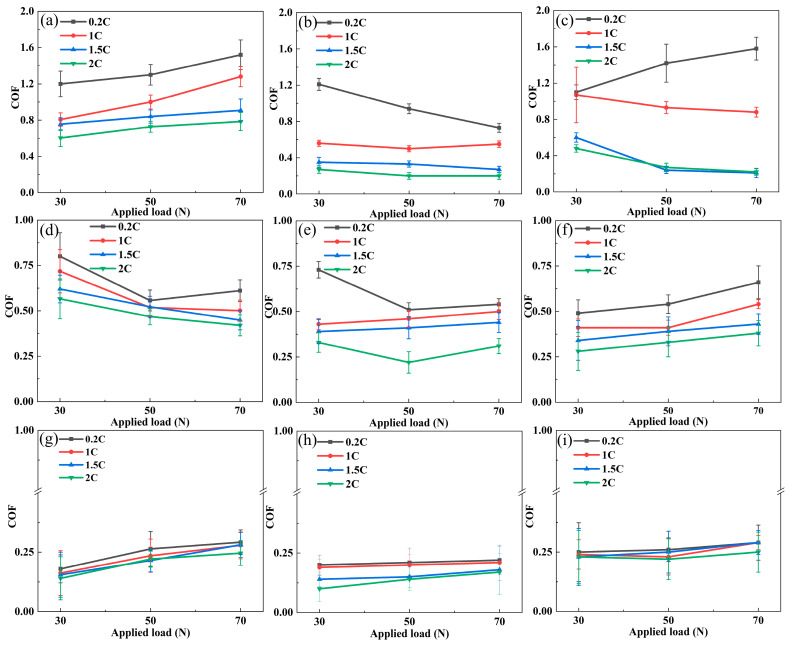
The relationship between the average friction coefficient and the applied load of the 0.2C, 1C, 1.5C, and 2C alloys in (**a**,**d**,**g**) dry wear and (**b**,**e**,**h**) H_2_SO_4_ and (**c**,**f**,**i**) HCl aqueous solutions under different sliding speeds: (**a**–**c**) 75, (**d**–**f**) 150, and (**g**–**i**) 225 mm/s.

Figure 5 shows the average friction coefficient curves of the 0.2C, 1C, 1.5C, and 2C alloys as a load function at different sliding rates. Figure 5 (a, d and, g), Figure 5 (b, e and, h), and Figure 5 (c, f and, i), respectively, correspond to dry wear conditions and tribocorrosion in H_2_SO_4_ and HCl. When the sliding rate is 75 mm/s, the COF gradually increases with the load under dry wear conditions, and the load increases the contact area, causing the friction resistance to increase. The COF in H_2_SO_4_ gradually decreases with load, and the COF in HCl decreases with load, except for the 0.2C alloy. When the sliding rate is 150 mm/s, the COF decreases with load in dry wear and H_2_SO_4_ tribocorrosion. The former is due to the lubrication effect of the alloy surface oxide, and the latter is the lubrication effect of the H_2_SO_4_ aqueous solution. The COF shows an increasing trend with load in HCl tribocorrosion. When the sliding rate is 75 and 150 mm/s, the COF decreases with the increase of alloy C content in the three wear states. This is because the rise of carbides reduces the actual contact area between the alloy and its counterpart. The COF in the H_2_SO_4_ solution is lower than that in the HCl solution, indicating that the former has a better lubrication effect. When the sliding rate is 225 mm/s, the COF does not change with the alloy’s load and C content. It is speculated that the wear mechanism has changed at this sliding rate compared with 75 and 150 mm/s.

**Figure 6:**
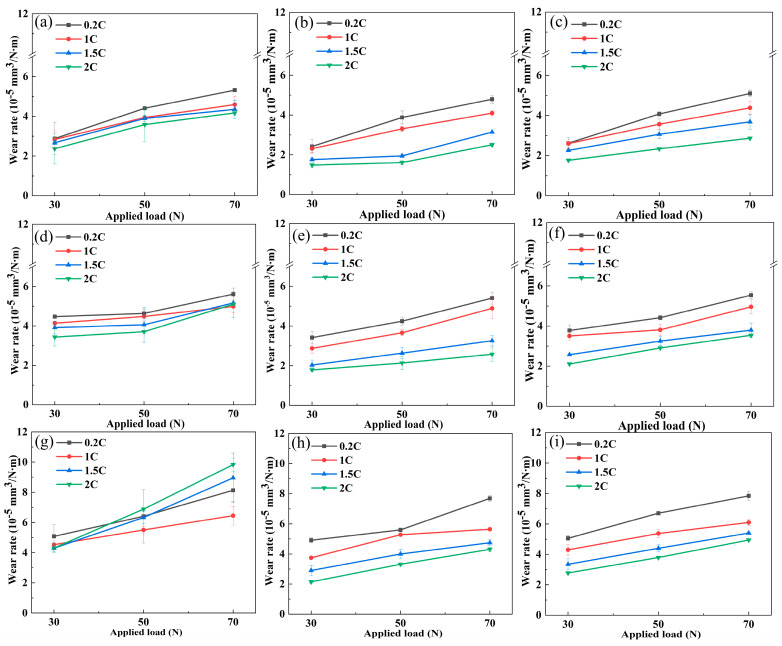
The relationship between the wear rate and the applied load of the 0.2C, 1C, 1.5C, and 2C alloys in (**a**,**d**,**g**) dry wear and (**b**,**e**,**h**) H_2_SO_4_ and (**c**,**f**,**i**) HCl aqueous solutions under different sliding speeds: (**a**–**c**) 75, (**d**–**f**) 150, and (**g**–**i**) 225 mm/s.

Figure 6 shows the wear rate curves of the 0.2C, 1C, 1.5C, and 2C alloys as the load changes at different sliding rates. Figure 6 (a, d and, g), Figure 6 (b, e and, h), and Figure 6 (c, f and, i), respectively, correspond to dry wear conditions and tribocorrosion in H_2_SO_4_ and HCl. At all sliding rates, the wear rate gradually increases with load, which increases the contact area and results in increased material removal. When the sliding rate is 75 mm/s, the alloy wear rate decreases with the C content and is inversely proportional to the Vickers hardness. When the sliding rate is 225 mm/s, the alloy wear rate increases with the C content, indicating that the wear mechanism has changed. The wear rates of tribocorrosion in H_2_SO_4_ and HCl are lower than dry wear. This is due to the lubrication and load distribution effects of the aqueous solution. The wear rate in H_2_SO_4_ is lower than that in HCl. This is consistent with the trend of the friction coefficient in Figure 5, indicating that the lubrication effect of H_2_SO_4_ is better than that of HCl.

### 3.4. Dry Sliding and Tribocorrosion Behavior of the NiCoCrMoCu-xC Alloys under Various Loads

**Figure 7:**
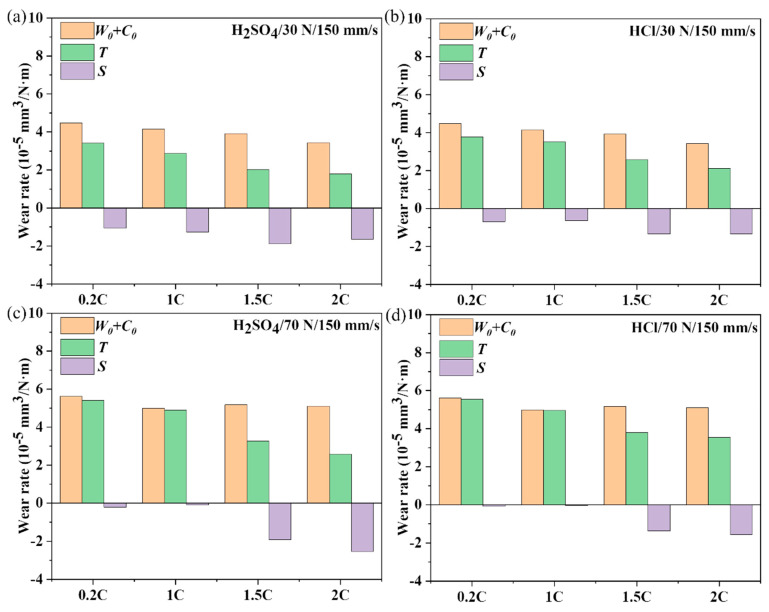
The contribution of mechanical wear (*W*_0_), corrosion (*C*_0_), and synergistic effects of corrosion and wear (*S*) on the tribocorrosion wear rate (*T*) of the 0.2C, 1C, 1.5C, and 2C alloys in (**a**,**c**) H_2_SO_4_ and (**b**,**d**) HCl aqueous solutions under (**a**,**b**) 30 and (**c**,**d**) 70 N loads.

According to Formula (2), the wear rate of the tribocorrosion of the 0.2C, 1C, 1.5C, and 2C alloys in H_2_SO_4_ and HCl was divided into three parts: *W*_0_, *C*_0_, and *S*, and the results are shown in Figure 7. Overall, the tribocorrosion wear rate *T* of the alloy is less than the mechanical wear rate *W*_0_ plus the corrosion rate *C*_0_ of the alloy; that is, the synergistic effect of corrosion and wear can reduce the wear rate of the alloy (*S* < 0). As the load increases, the antagonistic effect of the 0.2C and 1C alloys decreases, while that of the 1.5C and 2C alloys increases. The antagonistic effect on corrosion and wear of the 1.5C and 2C alloys is greater than that of the 0.2C and 1C alloys, regardless of loads, and the effect is more obvious in H_2_SO_4_ than in HCl. In liquid media, the thickness of the lubricating film between contact surfaces determines the lubrication effect to a certain extent. The film thickness $h_{min}$ can be calculated using the Hamrock–Dowson formula [47] as follows:

(3) $$h_{min}=\frac{3.63\alpha^{0.49}{\left(\eta U\right)}^{0.68}R^{0.466}}{E\prime^{0.117}P^{0.073}}\left(1-e^{-0.68k}\right)$$

where $h_{min}$ is the minimum hydrodynamic film thickness (μm), α is the material coefficient, η is the liquid viscosity (Pa·s), U is sliding speed (m/s), R is the equivalent radius of curvature (mm), E′ is the equivalent elastic modulus of the wear system (GPa), P is the load (N), and k is the ellipticity of the contact domain. It can be seen in Formula (3) that the thickness of the lubricating film is proportional to the viscosity of the liquid medium. The viscosities of H_2_SO_4_ [48] and HCl [49] at room temperature are 26.7 Pa·s and 10 mPa·s, respectively. The greater liquid viscosity is why the H_2_SO_4_ solution lubricates better than HCl.

**Figure 8:**
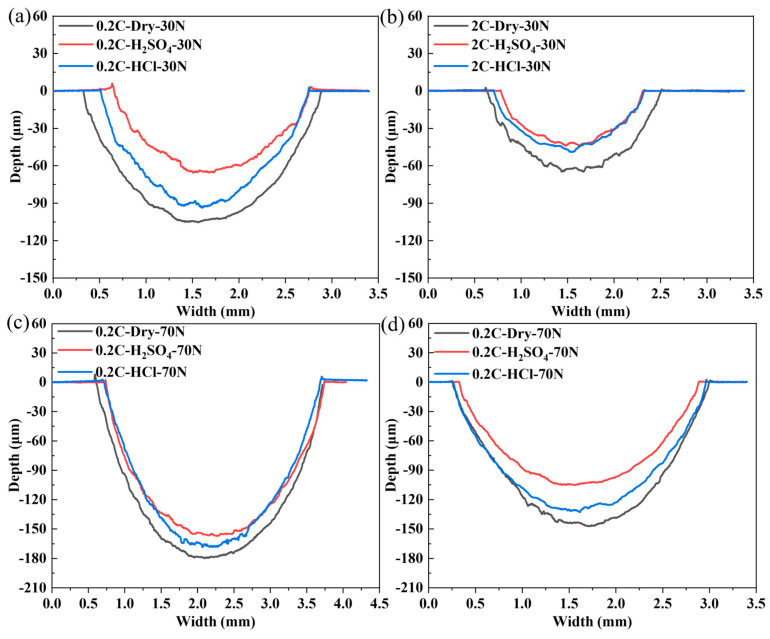
Two-dimensional width and depth results of (**a**,**c**) the 0.2C and (**b**,**d**) 2C alloys after dry sliding and tribocorrosion in H_2_SO_4_ and HCl aqueous solutions at (**a**,**b**) 30 and (**c**,**d**) 70 N.

Figure 8 shows the two-dimensional width and depth results of the 0.2C and 2C alloys after sliding under the load of 30 and 70 N, respectively. The wear cross-sectional area under dry wear is larger than that under tribocorrosion conditions, regardless of loads, which means that the lubricating effect of the solution on alloy wear is greater than the promoting effect of corrosion on wear, and the lubrication effect of the H_2_SO_4_ solution appears to be better than that of HCl. As the load increases, the cross-sectional area reduction in the 0.2C alloy shows a decreasing trend after corrosion wear compared with dry sliding wear, while the opposite trend is observed in the 2C alloy.

**Figure 9:**
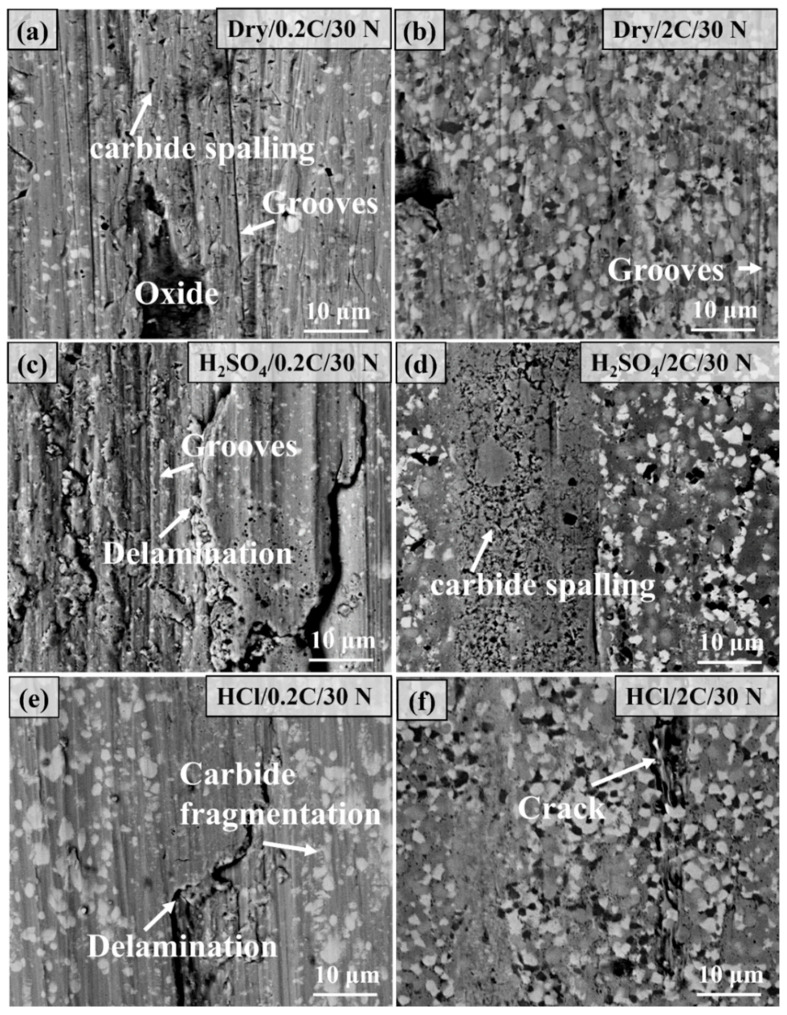
Comparison of the microstructure morphology of the wear surface of (**a**,**c**,**e**) the 0.2C and (**b**,**d**,**f**) 2C alloys under dry wear and (**c**,**d**) H_2_SO_4_ and (**e**,**f**) HCl aqueous solutions at 30 N and 75 mm/s parameters.

Figure 9 shows the comparison of the microstructure of the wear surface of the 0.2C and 2C alloys after dry sliding and tribocorrosion under a 30 N load. The main characteristics of the wear surface of the 0.2C alloy under dry wear conditions are grooves, oxidation, and carbide spalling, which are typical abrasive wear characteristics. Only slight scratch characteristics were observed in the 2C alloy, and the wear mechanism was abrasive wear. After tribocorrosion in the H_2_SO_4_ solution, the surface characteristics of the 0.2C alloy are grooves and delamination, which are typical delamination wear characteristics. The heat generated during the wear process is taken away by the solution, and the oxidation phenomenon disappears. The lubricating effect of the aqueous solution causes the surface to be uniformly pressured and work hardening on the alloy’s surface. Under repeated loads, the work-hardened layer generates cracks and expands to form massive wear debris, showing delamination characteristics. The surface characteristics of the 0.2C alloy after tribocorrosion in the HCl solution are similar to those in H_2_SO_4_, and carbide fragmentation was also observed, which is due to the fact that the HCl solution is not as lubricating as H_2_SO_4_, and the alloy surface is subjected to greater pressure. The wear surface of the 2C alloy after corrosion wear is smooth, and cracks are observed on the surface of the alloy in HCl.

**Figure 10:**
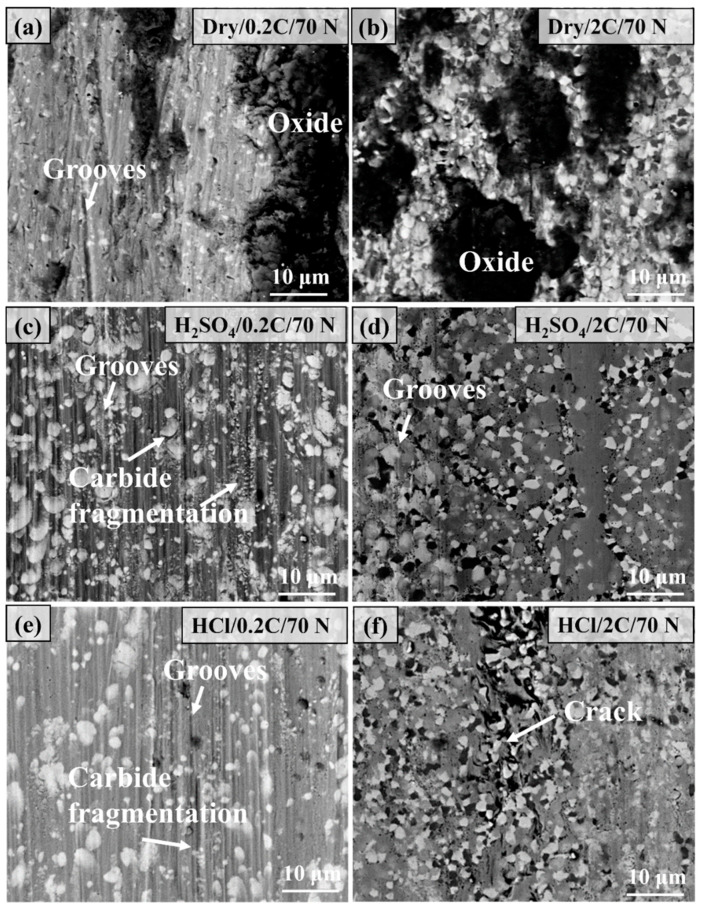
Comparison of the microstructure morphology of the wear surface of (**a**,**c**,**e**) the 0.2C and (**b**,**d**,**f**) 2C alloys after (**a**,**b**) dry sliding and tribocorrosion in (**c**,**d**) H_2_SO_4_ and (**e**,**f**) HCl aqueous solutions at 70 N and 75 mm/s.

Figure 10 shows the comparison of the microstructure of the wear surface of the 0.2C and 2C alloys after dry sliding and tribocorrosion under a 70 N load. Under dry sliding conditions, the depth and width of the groove on the surface of the 0.2C alloy are larger than that under 30 N, and the surface oxide coverage area increases. The wear mechanism is abrasive wear and oxidative wear. The surface of the 2C alloy is covered with a large number of oxides, and the main wear mechanism is oxidative wear. After tribocorrosion in H_2_SO_4_, the main characteristics of the wear surface of the 0.2C alloy are grooves and carbide fragmentation, and the main wear mechanism is abrasive wear. Compared with the 30 N load, the increase in load causes the contact area to increase and the lubricating film thickness to decrease, and the response of the wear surface changes from local cracking to carbide fragmentation. The degree of abrasive wear of the 2C alloy is reduced under the same load due to the higher hardness and yield strength. The increase in carbide content increases the bearing capacity of the alloy surface and eliminates the phenomenon of carbide fragmentation. The reason for local cracking in HCl is due to its relatively poor lubrication effect.

### 3.5. Wear and Tribocorrosion Behavior of the NiCoCrMoCu-xC Alloys under Various Sliding Speeds

**Figure 11:**
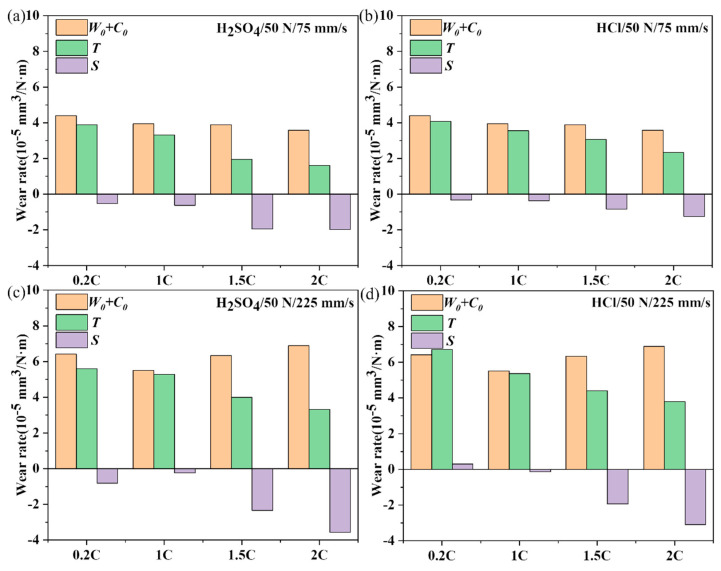
The contribution of mechanical wear (*W*_0_), corrosion (*C*_0_), and synergistic effects of corrosion and wear (*S*) on the tribocorrosion wear rate (*T*) of the 0.2C, 1C, 1.5C, and 2C alloys in (**a**,**c**) H_2_SO_4_ and (**b**,**d**) HCl aqueous solutions under (**a**,**b**) 75 and (**c**,**d**) 225 mm/s.

Figure 11 shows the comparison of dry sliding wear and tribocorrosion wear rates of the 0.2C and 2C alloys at sliding rates of 75 and 225 mm/s. The antagonistic effect of corrosion wear decreased in the 0.2C and 1C alloys but increased in the 1.5C and 2C alloys. The antagonistic effect of tribocorrosion decreased in the 0.2C and 1C alloys but increased in the 1.5C and 2C alloys. According to Formula (3), the thickness of the lubricating film increases with the sliding rate. The abnormal phenomena of the 0.2C and 1C alloys indicate that the wear mechanism may change with the sliding rate.

**Figure 12:**
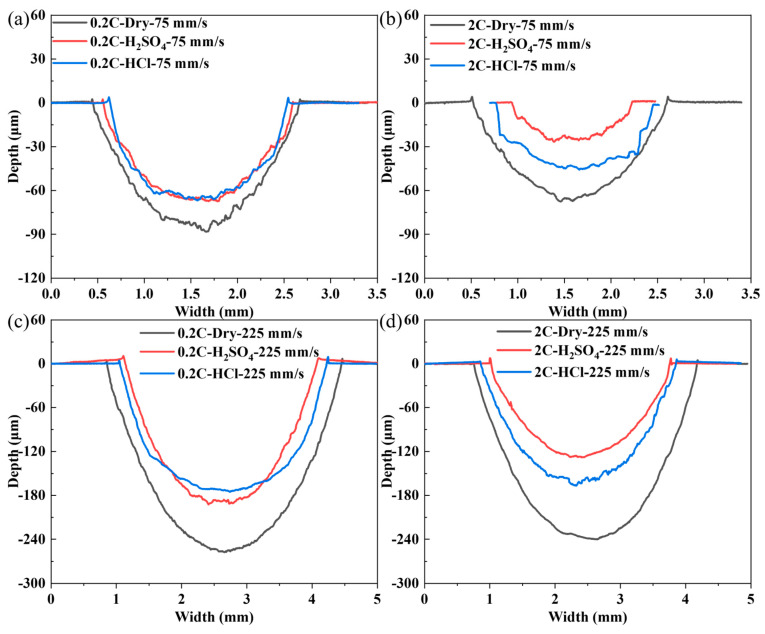
Two-dimensional width and depth results of (**a**,**c**) the 0.2C and (**b**,**d**) 2C alloys after dry sliding and tribocorrosion in H_2_SO_4_ and HCl aqueous solutions at (**a**,**b**) 75 and (**c**,**d**) 225 mm/s.

Figure 12 shows the two-dimensional width and depth results of the 0.2C and 2C alloys after sliding under sliding speeds of 75 and 225 mm/s, respectively. The wear cross-sectional area under dry wear is larger than that under tribocorrosion conditions, regardless of sliding speed. In the 2C alloys, the tribocorrosion antagonistic effect has obvious medium dependence, and the effect is stronger in H_2_SO_4_ than in HCl. The wear cross-sectional areas of the 0.2C alloy after tribocorrosion in H_2_SO_4_ and HCl are almost the same.

**Figure 13:**
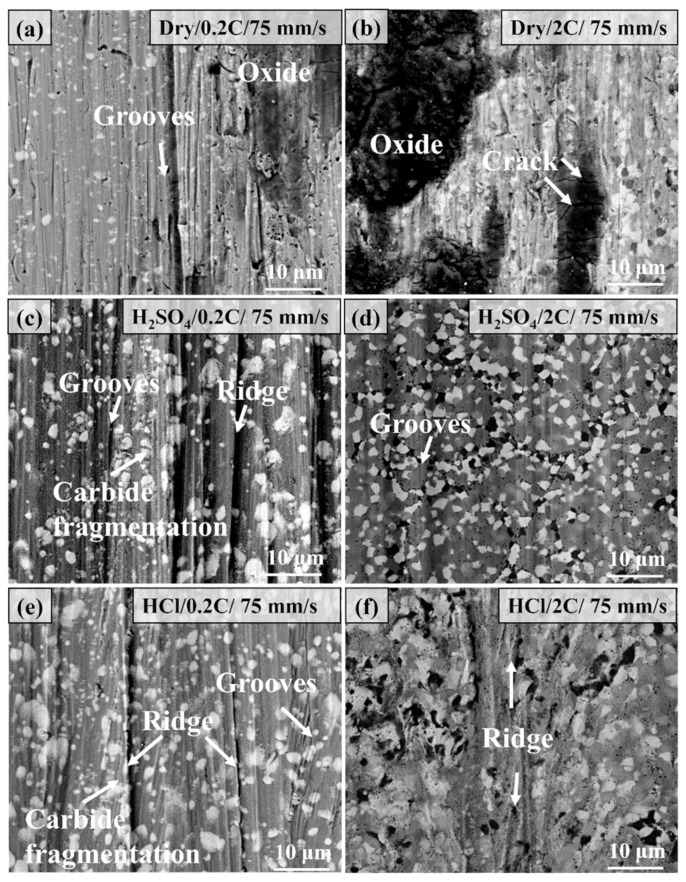
Comparison of the microstructure morphology of the wear surface of (**a**,**c**,**e**) the 0.2C and (**b**,**d**,**f**) 2C alloys after (**a**,**b**) dry sliding and tribocorrosion in (**c**,**d**) H_2_SO_4_ and (**e**,**f**) HCl aqueous solutions at 50 N and 75 mm/.

Figure 13 shows the wear surface morphology of the 0.2C and 2C alloys after dry sliding and tribocorrosion at a sliding rate of 75 mm/s. Under dry sliding wear, the wear surface of the 0.2C alloy is characterized by grooves of varying depths and a small amount of oxide adhesion, and the wear mechanism is abrasive wear. A large amount of oxide adhesion and slight scratches were observed on the worn surface of the 2C alloy; many cracks can be observed on the oxide surface, and the wear mechanism was oxidative wear. A large number of grooves and ridge features were observed on the surface of the 0.2C alloy after tribocorrosion in H_2_SO_4_, and carbide fragmentation on the worn surface was also observed, which are typical abrasive wear features. Only slight scratching was observed on the wear surface of the 2C alloy. The main feature of the surface of the 2C alloy is raised ridges, which is caused by the extrusion effect of carbides in the alloy on the matrix.

**Figure 14:**
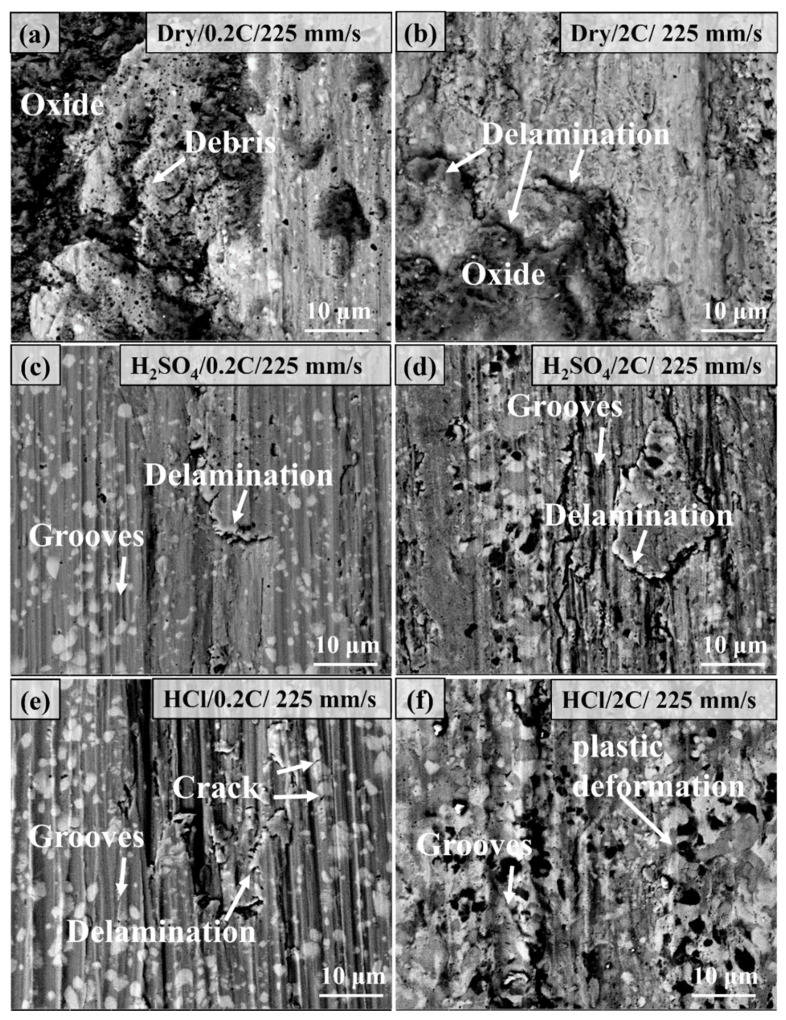
Comparison of the microstructure morphology of the wear surface of (**a**,**c**,**e**) the 0.2C and (**b**,**d**,**f**) 2C alloys after (**a**,**b**) dry sliding and tribocorrosion in (**c**,**d**) H_2_SO_4_ and (**e**,**f**) HCl aqueous solutions at 50 N and 225 mm/s.

Figure 14 shows the microstructure morphology of the wear surface of the 0.2C and 2C alloys after dry sliding wear and tribocorrosion at a sliding rate of 225 mm/s. The main characteristics of the 0.2C surface under dry wear conditions are the accumulation of wear debris and the adhesion of oxides and grooves almost disappears compared with 75 mm/s, this is a typical adhesion wear feature. The heat generated by the high sliding rate softens and oxidizes the wear debris on the wear surface and is cold welded to the surface under the repeated action of the counterpart. The surface of the 2C alloy shows obvious delamination and oxidation characteristics, which are typical delamination wear characteristics; among them, the outermost layer has the most obvious degree of oxidation, and the material is removed layer by layer through wear and cracking. The surface characteristics of both the 0.2C and 2C alloys after tribocorrosion in H_2_SO_4_ are grooves and delaminations. The increase in the sliding rate increases the frequency of action on the alloy surface per unit time, resulting in the work hardening of the surface and cracking. The wear mechanism of the 0.2C alloy in HCl is abrasive wear and is represented by grooves and delamination wear caused by cracking. The wear surface of the 2C alloy shows wide grooves and carbide aggregation on the surface, which is caused by the plastic deformation of the matrix around the carbides.

### 3.6. Worn Surface Characterization of the 0.2C and 2C Alloys in Various Conditions

Table 4 summarizes the roughness of the wear surface of the 0.2C and 2C alloys after wear under different conditions and parameters. Overall, the roughness of the 2C alloy is lower than that of the 0.2C alloy under all wear conditions and parameters, indicating that the increase in carbides helps maintain the integrity of the wear surface of the alloy during wear. The surface roughness is the lowest after tribocorrosion in H_2_SO_4_ and the highest under dry wear conditions, which benefits from the lubrication of the aqueous solution.

**Figure 15:**
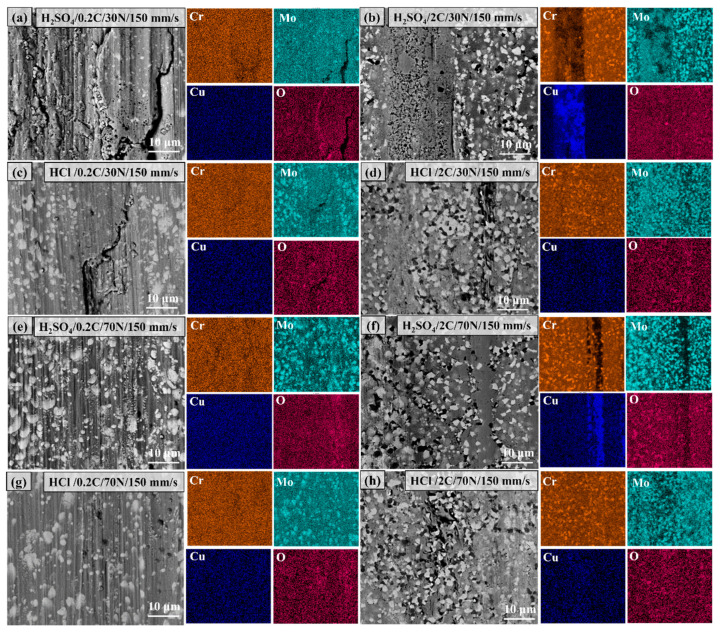
The morphology and elemental distribution of the worn surface of (**a**,**c**,**e**,**g**) the 0.2C and (**b**,**d**,**f**,**h**) 2C alloys subjected to tribocorrosion in (**a**,**b**,**e**,**f**) H_2_SO_4_ and (**c**,**d**,**g**,**h**) HCl under (**a**–**d**) 30 and (**e**–**h**) 70 N loads.

Figure 15 shows the morphology and elemental distribution of the worn surface of the 0.2C and 2C alloys subjected to tribocorrosion in H_2_SO_4_ and HCl under 30 and 70 N loads. The morphology of the wear surface has been analyzed above, and here we focus on analyzing the distribution of corrosion-resistant elements, Cr, Mo, and Cu. The Cr and Cu elements are evenly distributed on the surface of the 0.2C alloy, and the Mo element is enriched in the form of carbides. After tribocorrosion under 30 and 70 N loads in H_2_SO_4_, the 2C alloy surface showed obvious enrichment of the Cu element. The enrichment of the Cu element may be due to the following three reasons. 1. Element segregation caused by severe plastic deformation. Feng et al. [50] observed obvious grain boundary segregation of the Cu element in the Al-Cu alloy after high-pressure torsional deformation. 2. The dissolution and re-precipitation behavior of the Cu element in the H_2_SO_4_ solution. Zhang et al. [24] and Yamanaka et al. [51] observed the enrichment phenomenon of the Cu alloy on the surface in the sulfuric acid corrosion experiment of the carbide-strengthened Fe-16Cr-3W-1C-xCu alloy. Cu significantly improved the corrosion resistance of the alloy. 3. Preferential corrosion of the matrix and precipitation of Cu particles caused by contact stress. Zhu et al. [29] observed the enrichment of Cu particles on the surface of the wear surface under stress in the corrosion and wear experiment of the Monel400 (Ni-30Cu) alloy in seawater. The lubrication effect of Cu significantly reduces the friction coefficient and the wear rate of the alloy. There is no obvious enrichment of Cu on the surface of the 2C alloy after corrosion and wear in HCl. The enriched position of the O element in the wear surface of the 2C alloy after corrosion wear coincides with the position of the Cr_23_C_6_ carbide. It is speculated that Cr_2_O_3_ is formed on the surface of Cr_23_C_6_. Webb et al. [52] pointed out that carbide oxidation occurs by the diffusion of oxygen through the surface oxide layer down to the oxide–carbide interface. Matthews et al. [53] found in high-temperature oxidation experiments of Cr_3_C_2_/NiCr composite coatings that chromium carbide can be oxidized through a gradual decarburization mechanism to form Cr_2_O_3_, and Cr_3_C_2_ is oxidized to Cr_7_C_3_, Cr_23_C_6,_ and Cr_2_O_3_ in sequence. The enrichment of Cu [54] and the presence of Cr_2_O_3_ [55] can not only reduce the corrosion of the alloy but also reduce the wear rate of the alloy through lubrication.

**Figure 16:**
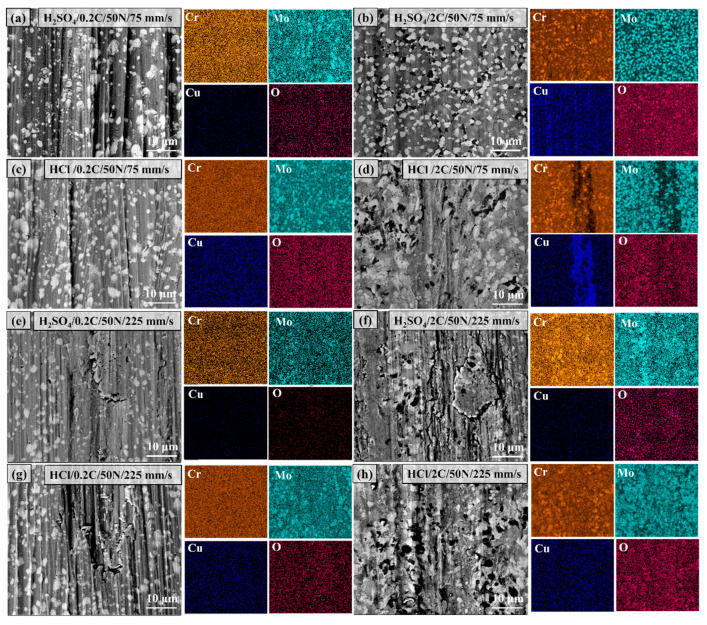
The morphology and elemental distribution of the worn surface of (**a**,**c**,**e**,**g**) the 0.2C and (**b**,**d**,**f**,**h**) 2C alloys subjected to tribocorrosion in (**a**,**b**,**e**,**f**) H_2_SO_4_ and (**c**,**d**,**g**,**h**) HCl under (**a**–**d**) 75 and (**e**–**h**) 225 mm/s sliding speed.

Figure 16 shows the morphology and elemental distribution of the worn surface of the 0.2C and 2C alloys subjected to tribocorrosion in H_2_SO_4_ and HCl under sliding speeds of 75 and 225 mm/s. No segregation of the Cr, Mo, and Cu elements was observed after the wear of the 0.2C alloy at both sliding rates. The enrichment of the Cu element was observed on the surface of the 2C alloy after tribocorrosion in H_2_SO_4_ and HCl at a sliding rate of 75 mm/s, which was distributed in a chain shape in H_2_SO_4_. It is distributed in strips in HCl and the enriched area is the area of severe plastic deformation on the surface, indicating that plastic deformation can promote the segregation of Cu. No segregation of Cu was observed on the surface of the 2C alloy after tribocorrosion at a sliding rate of 225 mm/s. This is due to the fatigue damage of the surface wear layer caused by the high sliding rate. The oxidation of Cr_23_C_6_ was observed on the wear surface of the 2C alloy at all sliding rates.

### 3.7. Optimization of the COF and Wear Rate Using the Response Surface Model

**Figure 17:**
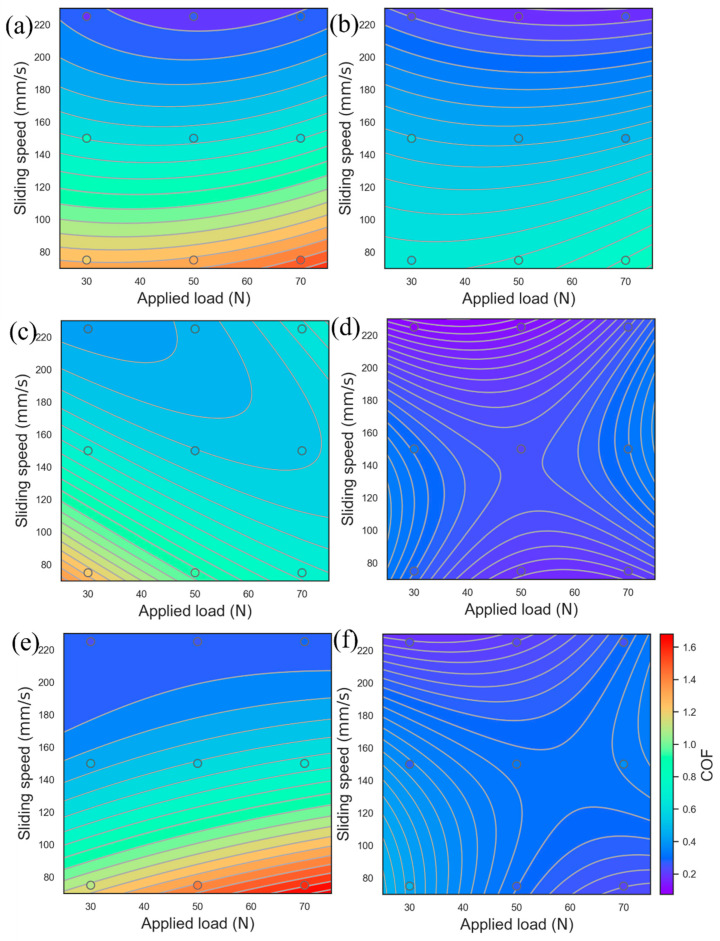
Evolution of the COF concerning the applied load and sliding speed for (**a**,**b**) dry sliding and tribocorrosion in (**c**,**d**) H_2_SO_4_ and (**e**,**f**) HCl of (**a**,**c**,**e**) the 0.2C and (**b**,**d**,**f**) 2C alloys using response surface analysis.

**Figure 18:**
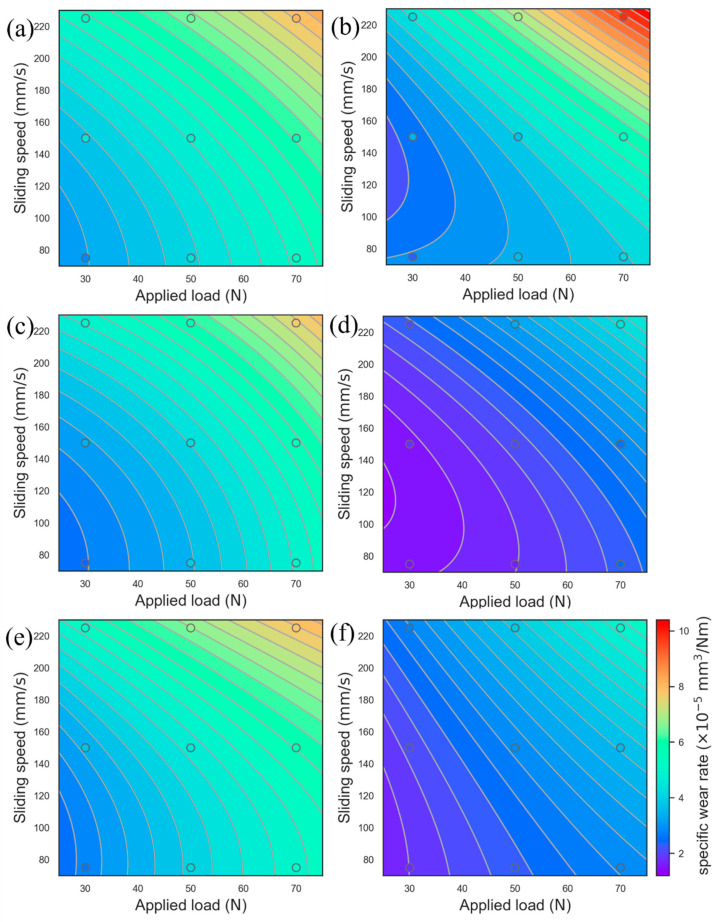
Evolution of the wear rate concerning the applied load and sliding speed for (**a**,**b**) dry sliding and tribocorrosion in (**c**,**d**) H_2_SO_4_ and (**e**,**f**) HCl of (**a**,**c**,**e**) the 0.2C and (**b**,**d**,**f**) 2C alloys using response surface analysis.

Figure 17 and Figure 18 show the COF and wear rate fitting diagrams obtained under different wear states and parameters using the response surface analysis method (RSM) [56,57]. The variables are loads and sliding speeds, and the solid points are experimental data. The multiple quadratic regression equation used for fitting is shown in Formula (4) as follows:

(4) y=b1+b2·x1+b2·x2+b3·x1·x1+b4·x2·x2+b5·x1·x2

where b1, b2, b3, b4, and b5 are the fitting coefficients, x1, and x2 are the load and sliding rate, respectively, and y is the COF or wear rate.

Figure 17 and Figure 18 show the COF and wear rate behavior of the 0.2C and 2C alloys in dry sliding and tribocorrosion experiments. Under dry sliding conditions, the COF behavior in the 0.2C and 2C alloys shows obvious sliding rate dependence, and the wear rate behavior shows load and sliding rate dependence. When sliding at 75 mm/s, the surface degradation form of the 0.2C and 2C alloys is abrasive wear. The yield strength of the 0.2C alloy is lower than that of the 2C alloy under the same load, and the depth of action of the friction pair on the surface of the 0.2C alloy is greater, leading to an increase in the COF. Under load, the surface of the 0.2C alloy appears with the characteristics of grooves and carbide spalling. There is only slight scratching on the surface of the 2C alloy; less material is removed, and the wear rate is low. As the sliding rate increases to 150 mm/s, the surface oxides of the 0.2C and 2C alloys increase, and the COF decreases. When the sliding rate increases to 225 mm/s, plastic deformation occurs on the surface of the 0.2C and 2C alloys due to the softening of the matrix [39], and the COF further decreases. The softened matrix cracks and generates massive wear debris after repeated cold work hardening, and the wear rate increases significantly.

Under tribocorrosion conditions, the solution lubricates the wear surface and distributes the load evenly [58]. The COF behavior and wear rate behavior of the 0.2C alloy did not change significantly compared with dry sliding conditions, while the 2C alloy showed an obvious tribocorrosion antagonistic effect; that is, the COF and wear rate were significantly reduced. This is consistent with the experimental results in Figure 7 and Figure 11. During the sliding process, the solution can lubricate the worn surface and evenly distribute the load [59]. Due to the low yield strength and the high work hardening ability of the 0.2C alloy, the surface shows the characteristics of delamination wear caused by fatigue cracks, and the wear rate does not decrease significantly compared with the dry sliding wear state. During the tribocorrosion process, Cu enrichment and Cr_23_C_6_ surface oxidation occurred on the 2C alloy surface, which enhanced the lubrication effect during the sliding process, and the COF and wear rate decreased. The yield strength of the 2C alloy is higher than that of the 0.2C alloy; only slight scratch marks are left on the surface after sliding, and less material is removed.

## 4. Conclusions

In this study, we conducted tribocorrosion tests on the NiCoCrMoCu alloys with different carbide contents/types in two different acidic media (H_2_SO_4_ and HCl) under various external loading conditions (load and sliding speed). The dry sliding wear behavior of the same alloys was also studied to compare it with the tribocorrosion behavior. The main conclusions are summarized as follows:

1. The carbide content, Vickers hardness, and yield strength in the NiCoCrMoCu-xC alloy increase with the C content, whereas the alloy grain size is refined with the increase of carbide content.
2. Compared with dry sliding wear, the NiCoCrMoCu-xC alloys exhibit a lower wear rate in the tribocorrosion process due to the lubrication effect of the solution, regardless of wear parameters. The lubrication effect can be promoted by the enrichment of Cu elements and the Cr_2_O_3_ on the surface. In addition, the H_2_SO_4_ solution has a better lubricating effect than HCl because of its higher viscosity.
3. A general antagonistic effect was observed in the tribocorrosion of the NiCoCrMoCu-xC alloy. As the load and sliding rate increased, the antagonistic effect of the 0.2C and 1C alloys decreased because the destruction of the integrity of the wear surface leads to the disappearance of the Cu-rich zone and Cr_2_O_3_.
4. The COF and wear rate under different loads and sliding rates were analyzed using the response surface analysis method. It was found that the COF mainly showed dependence on the sliding rate, while the wear rate showed dependence on load and the sliding rate.

## Figures and Tables

**Table 1:** Nominal composition of the 0.2C, 1C, 1.5C, and 2C alloys (wt.%).

Nominations	Ni	Co	Cr	Mo	Cu	W	V	Si	Ti	Mn	C
0.2C	Bal.	30	17	16	2	4	—	—	—	—	0.2
1C	Bal.	20	20	16	1.5	2	2	0.25	0.25	0.5	1
1.5C	Bal.	20	20	16	1.5	3	3	0.375	0.375	0.5	1.5
2C	Bal.	20	20	16	1.5	4	4	0.5	0.5	0.5	2

**Table 2:** Phase fractions and average particle size of the Ni_3_Mo_3_C, Cr_23_C_6,_ and V_2_C carbides.

Alloys		Ni_3_Mo_3_C	Cr_23_C_6_	V_2_C	Area Fraction, %
0.2C	Phase fraction, %	9.38	—	—	9.5
	Size, μm	1.03	—	—	—
1C	Phase fraction, %	25.0	10.0	—	35
	Size, μm	1.39	1.77	—	—
1.5C	Phase fraction, %	23.5	5.8	11.8	41.1
	Size, μm	1.42	0.67	0.7	—
2C	Phase fraction, %	17.3	6.1	17.8	41.2
	Size, μm	1.08	0.63	0.73	—

**Table 3:** Compressive properties and maximum contact stress [41] at 30, 50, and 70 N loads for the 0.2C, 1C, 1.5C, and 2C alloys.

Alloys	Yield Strength (MPa)	Ultimate Compressive Strength (MPa)	Maximum Contact Stress (MPa)		
			30 N	50 N	70 N
0.2C	698	2287	2077	2463	2755
1C	978	1873	2114	2507	2804
1.5C	1166	2007	2141	2538	2839
2C	1335	2088	2163	2565	2870

**Table 4:** The roughness of the wear surface of the 0.2C and 2C alloys after wear under different conditions and parameters.

Roughness/μm	0.2C-Dry	0.2C-H_2_SO_4_	0.2C-HCl	2C-Dry	2C-H_2_SO_4_	2C-HCl
30 N/75 mm/s	0.228	0.109	0.138	0.144	0.074	0.166
70 N/75 mm/s	0.282	0.045	0.176	0.150	0.062	0.119
75 mm/s/50 N	0.175	0.085	0.136	0.267	0.031	0.042
225 mm/s/50 N	0.185	0.127	0.142	0.256	0.14	0.057

## Data Availability

The data presented in this study are available on request from the corresponding author.

## Nomenclature

*ω*	wear rate(10^−5^ mm^3^/N·m)
*V*	wear volume loss (mm^3^)
*L*	the sliding distance (m)
*P*	the applied load (N)
*T*	wear rate caused by tribocorrosion (10^−5^ mm^3^/N·m)
*W*_0_	wear rate caused by dry sliding wear (10^−5^ mm^3^/N·m)
*C*_0_	wear rate caused by immersion corrosion (10^−5^ mm^3^/N·m)
*S*	wear rate caused by the synergistic effect of corrosion and wear (10^−5^ mm^3^/N·m)
*H*_min_	the minimum hydrodynamic film thickness (μm)
*α*	constant
*η*	the liquid viscosity (Pa·s)
*U*	sliding speed (m/s)
*R*	equivalent radius of curvature(mm)
E′	the equivalent elastic modulus of the wear system (GPa)
*k*	the ellipticity of the contact domain
